# Editorial: Spotlight on artificial intelligence in experimental pharmacology and drug discovery

**DOI:** 10.3389/fphar.2023.1261141

**Published:** 2023-08-28

**Authors:** Verena Schöning, Amit Khurana, Aleksandra Karolak

**Affiliations:** ^1^ Clinical Pharmacology and Toxicology, Department of General Internal Medicine, Inselspital, Bern University Hospital, University of Bern, Bern, Switzerland; ^2^ Institute of Molecular Pathobiochemistry, Experimental Gene Therapy and Clinical Chemistry, University Hospital Aachen (UKA), RWTH University, Aachen, Germany; ^3^ Department of Machine Learning, Moffitt Cancer Center, Tampa, FL, United States

**Keywords:** artificial intelligence, pharmacology, drug discovery, pharmaceutical research, machine learning

The rapid advancement of artificial intelligence (AI) has revolutionized the field of experimental pharmacology and drug discovery. Many recent works have shed light on groundbreaking research and technological advancements in biomedical sciences and drug development. In this Research Topic, we bring to our readers’ attention six innovative articles that demonstrate the potential of AI in addressing critical challenges in biomedical fields. From identifying crucial kinases to generating medication guides, characterizing small-molecule effects, enabling early-stage drug development, and repurposing drugs for SARS-CoV-2, these articles highlight the transformative role and illuminate the potential of AI in reshaping the future of pharmaceutical research. They demonstrate how AI-driven approaches can address key challenges in the field and contribute to advancements in therapeutic development and drug discovery.

Gruver et al. highlight the evolving understanding of pro-angiogenic growth factors and their significance in tumor angiogenesis. By presenting a novel approach to identify critical kinases involved in endothelial cell proliferation, the authors provide new insights into the development of comprehensive anti-angiogenic therapies. This research contributes to the ongoing battle against cancer by expanding our knowledge of pro-angiogenic signaling pathways. Meyer et al. focus on the regulatory medical writing, which has faced increased importance due to the rising number of new drug applications. The application of natural language processing techniques to generate medication guides from existing drug label information offers a promising solution. This approach brings potential to modernize medical writing processes, reduce manual labor, and enhance efficiency and accuracy in carrying critical information to healthcare professionals and patients. By integrating heterogeneous data into a human interactome network, Mangione et al. aim to understand drug behavior in biological systems via the enhanced Computational Analysis of Novel Drug Opportunities (CANDO) platform applied to mental disorders and cancer metastasis. CANDO allows for the integration of drug side effects, protein pathways, protein–protein interactions, protein–disease associations, and the Gene Ontology, complemented with the existing drug/compound, protein, and indication libraries. This broad characterization contributes to improved therapeutic candidate selection and enhances drug discovery while addressing two major challenges in therapeutic clinical trials: efficacy and safety. Barrett et al. explore the integration of AI into early-stage drug development. Recognizing the significance of target evaluation and understanding disease progression, the authors investigate how AI algorithms, combined with digital research environments, can effectively manage diversity and volume of data and predict outcomes. This integration offers promising opportunities to accelerate drug development while ensuring data security and intellectual property protection. Elkashlan et al. address the need to fight SARS-CoV-2 and discuss the frequently used databases and machine learning (ML) models utilized to target the virus. The authors discuss the features and limitations of the databases, while the ML models, including deep learning and conventional ML models, are discussed from the perspective of the underlying methodology and its recent applications in the prospective predictions of SARS-CoV-2 inhibitors. This approach has the potential to expedite drug discovery processes, providing effective solutions to the fight against emerging infectious diseases. Lastly, Ruatta et al. emphasize the critical challenge of identification of SARS-CoV-2 inhibition and the importance of computational tools having power to integrate, process, and analyze multiple data in a short time. However, the authors highlight serious risks of obtaining unrealistic results when the utilized models are not derived from trustworthy data and the resultant predictions lack validation through experimental evidence. This study reaffirms the fundamental principle of “garbage in, garbage out” and highlights the importance of data quality and experimental validation in *in silico* drug discovery.

Jointly, these articles demonstrate the potential of AI for addressing various aspects of pharmacology and drug discovery from identifying crucial kinases to restructuring medical writing processes, characterizing small-molecule effects, facilitating early-stage drug development, repurposing drugs for emerging diseases, and improving virtual screening approaches. With the emergence of AI-aided discoveries offering significant prospects in development therapeutics that are both safe and effective, AI-driven approaches are beginning to transform the pharmaceutical industry.

